# Supplementary material for: Auditory and Visual Response Inhibition in Children with Bilateral Hearing Aids and Children with ADHD
Source: Brain Sci. 2020 May 18;10(5):307. doi: 10.3390/brainsci10050307 (PMC7287647; doi:10.3390/brainsci10050307)
Supplement: Supplementary file 1 [file brainsci-10-00307-s001.zip › SupplementaryMaterial_BrainSciences_LB.docx]

Supplementary Material

**Table S1.** Hearing aid (HA) information for the HA group.

| **HA user** | **Age at HL diagnosis: (years of age)** | **Age at first HA fitting:  *M* (*SD*) (years of age)** | **HA use** | | **Unaided pure-tone average threshold**  **(0.5-4 kHz)** | |
| --- | --- | --- | --- | --- | --- | --- |
|  |  |  | **School day (h)** | **Out of school day (h)** | **Left ear**  **(dB)** | **Right ear**  **(dB)** |
| A | For 9 children < 2.5,  for 4 children between 3-4, and for 2 children between 5-9 years of age | 2.85 (2.29) | 13 | 13 | 55 | 51.9 |
| B |  |  | 11 | 10 | 63.5 | 61 |
| C |  |  | 14 | 14 | 65.5 | 73 |
| D |  |  | 12 | 12 | 49 | 44.5 |
| E |  |  | 15 | 12 | 50 | 50.5 |
| F |  |  | 10 | 8 | 36.5 | 35.5 |
| G |  |  | 12.5 | 11 | 44.5 | 43 |
| H |  |  | 13 | 15 | 75 | 75 |
| I |  |  | 12 | 14 | 62 | 57 |
| J |  |  | 14.5 | 14.5 | 98 | 97 |
| K |  |  | 14.5 | 14.5 | 70 | 72 |
| L |  |  | 14 | 16 | 96 | 80 |
| M |  |  | 12 | 12 | 66 | 75 |
| N |  |  | 15.5 | 15.5 | 105 | 91 |
| O |  |  | 12.5 | 12.5 | 71 | 76 |

Note: The demographic data for the HA group is listed. For information on age, descriptive information and a group average are presented to ensure anonymity. The audiometry indicated an average hearing loss in children with HAs as follows: right ear: 55.33 dB for 0.25 kHz, 59 dB for 0.5 kHz, 63.5 for 1 kHz, 73.67 for 2 kHz, 69.46 for 3 kHz, 63 dB for 4 kHz, 66.43 for 6 kHz and 66.43 for 8 kHz; left ear: 51.33 dB for 0.25 kHz, 59.67 dB for 0.5 kHz, 65.67 for 1 kHz, 75.67 for 2 kHz, 68.08 for 3 kHz, 62.14 dB for 4 kHz, 67.14 for 6 kHz and 71.07 for 8 kHz. For an illustration of the audiometry see Figure S1. Abbreviations: HA, hearing aid/child (A-O) of the hearing aid group; M, mean; SD, standard deviation.


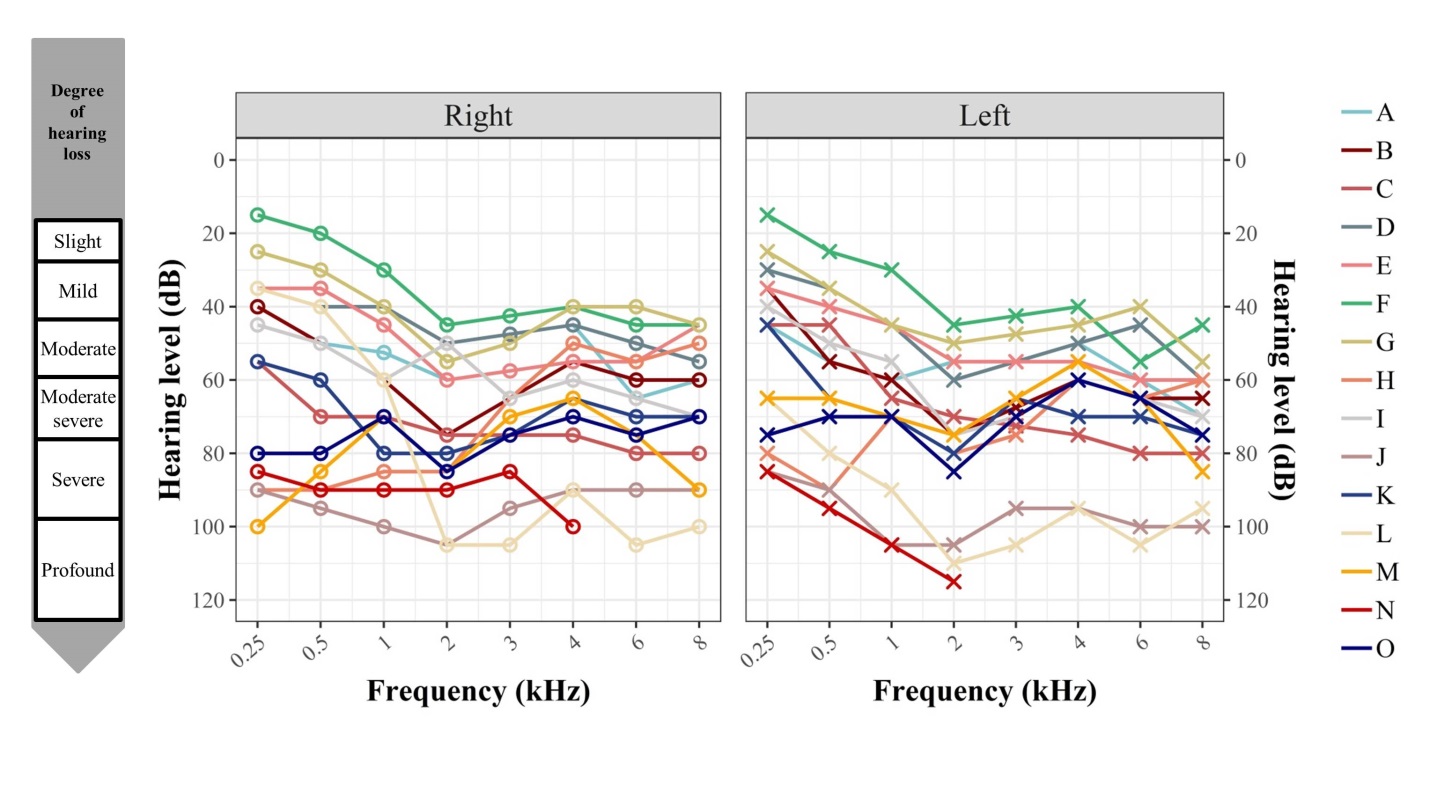


**Figure S1.** Unaided pure tone audiometry (air-conduction) of the right and left ear for each child (A-O) of the HA group.

**Table S2.** Uncorrected *t*-tests of the significant (*p* < 0.05) contrasts of ΔHbO in each condition against baseline.

|  | **Ch** | **Group** | **Brain region** | ***t*** | **df** | ***p*** |
| --- | --- | --- | --- | --- | --- | --- |
| **Visual** | P1 Ch 4 | ADHD | Left supramarginal gyrus | 3.53 | 13 | 0.004 |
|  | P1 Ch 7 | TD | Left DLPFC | -2.82 | 14 | 0.01 |
|  | P1 Ch 8 | TD | Left primary somatosensory cortex | 2.37 | 14 | 0.03 |
|  | P2 Ch1 | ADHD | Right supramarginal gyrus | 2.55 | 11 | 0.03 |
|  | P2 Ch2 | HA | Right pre/SMA | 2.43 | 10 | 0.04 |
|  | P2 Ch6 | HA | Right primary somatosensory cortex | 2.23 | 11 | 0.048 |
|  |  | ADHD |  | 2.75 | 13 | 0.02 |
|  | P2 Ch9 | ADHD | Right frontopolar cortex | 2.35 | 14 | 0.03 |
|  | P2 Ch10 | ADHD | Right primary auditory cortex | 2.80 | 13 | 0.02 |
|  | P2 Ch15 | TD | Right STG | 2.57 | 14 | 0.02 |
|  | P2 Ch13 | ADHD | Right frontopolar cortex | 2.39 | 12 | 0.03 |
|  | P2 Ch16 | TD | Right STG | -2.39 | 13 | 0.03 |
|  | P2 Ch17 | HA | Right IPFG | 3.43 | 9 | 0.008 |
|  | P2 Ch21 | TD | Right temporopolar cortex | -2.73 | 14 | 0.02 |
| **Auditory** | P1 Ch8 | ADHD | Left primary somatosensory cortex | 2.23 | 12 | 0.046 |
|  | P2 Ch1 | ADHD | Right supramarginal gyrus | 3.17 | 11 | 0.009 |
|  | P2 Ch5 | ADHD | Right supramarginal gyrus | 2.60 | 12 | 0.02 |
|  | P2 Ch6 | ADHD | Right primary somatosensory cortex | 4.17 | 13 | 0.001 |
|  | P2 Ch10 | ADHD | Right primary auditory cortex | 2.79 | 13 | 0.02 |
|  | P2 Ch18 | ADHD | Right frontopolar cortex | 2.30 | 14 | 0.04 |
|  | P2 Ch21 | TD | Right temporopolar cortex | -2.74 | 14 | 0.02 |

Abbreviations: HA, hearing aid; ADHD; attention deficit/hyperactivity disorder; TD, typically developing; P, probe; Ch, channel; IPFG, inferior prefrontal gyrus; STG, superior temporal gyrus; MTG, medial temporal gyrus; pre/SMA, pre-motor and supplementary motor area.

**Table S3.** Uncorrected *t*-tests of the significant (*p* < 0.05) contrasts of ΔHbR in each condition against baseline.

|  | **Ch** | **Group** | **Brain region** | ***t*** | **df** | ***p*** |
| --- | --- | --- | --- | --- | --- | --- |
| **Visual** | P1 Ch7 | HA | Left DLPFC | 2.31 | 11 | 0.04 |
|  |  | ADHD |  | 3.59 | 12 | 0.004 |
|  | P1 Ch8 | HA | Left primary somatosensory cortex | -2.25 | 11 | 0.046 |
|  | P1 Ch12 | ADHD | Left pre/SMA | 3.42 | 14 | 0.004 |
|  | P1 Ch16 | ADHD | Left pars opercularis | 3.05 | 12 | 0.01 |
|  | P1 Ch17 | ADHD | Left STG | 2.21 | 14 | 0.04 |
|  | P1 Ch18 | TD | Left STG | 2.62 | 13 | 0.02 |
|  | P2 Ch2 | HA | Right pre/SMA | -2.91 | 10 | 0.02 |
|  | P2 Ch9 | TD | Right frontopolar cortex | 2.24 | 14 | 0.04 |
|  | P2 Ch14 | TD | Right STG | 2.46 | 14 | 0.03 |
|  | P2 Ch16 | ADHD | Right STG | 3.42 | 12 | 0.03 |
|  | P2 Ch17 | HA | Right IPFG | -2.47 | 9 | 0.04 |
| **Auditory** | P1 Ch3 | ADHD | Left pre/SMA | 2.34 | 11 | 0.04 |
|  | P1 Ch7 | ADHD | Left DLPFC | 3.25 | 12 | 0.007 |
|  | P1 Ch16 | ADHD | Left pars opercularis | 3.42 | 12 | 0.005 |
|  | P1 Ch17 | TD | Left STG | -3.57 | 14 | 0.003 |
|  | P2 Ch3 | HA | Right DLPFC | -2.51 | 7 | 0.04 |
|  |  | ADHD |  | 2.99 | 12 | 0.01 |
|  | P2 Ch20 | HA | Right MTG | -2.30 | 10 | 0.04 |
|  |  | TD |  | -2.39 | 14 | 0.03 |

Abbreviations: HA, hearing aid; ADHD; attention deficit/hyperactivity disorder; TD, typically developing; P, probe; Ch, channel; IPFG, inferior prefrontal gyrus; STG, superior temporal gyrus; MTG, medial temporal gyrus; pre/SMA, pre-motor and supplementary motor area.


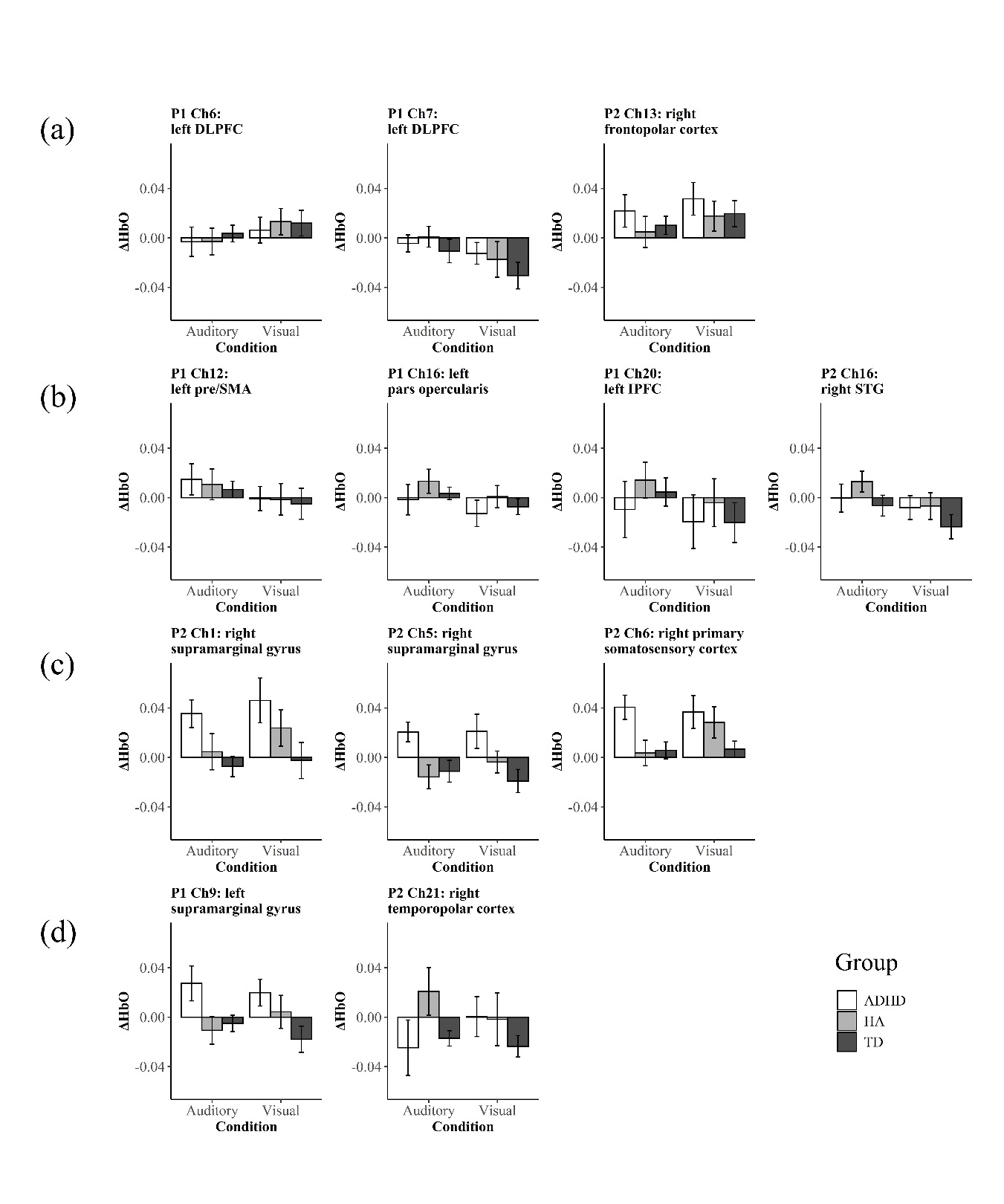


**Figure S2.** Effects of group (HA, ADHD, TD) and condition (auditory, visual) on ΔHbO. All effects of (a) Condition: Visual > Auditory, (b) Condition: Auditory > Visual, (c) Group and (d) Group-by-condition on ΔHbO are illustrated. Abbreviations: P, Probe set; Ch, channel; DLPFC, dorsolateral prefrontal cortex; pre/SMA, pre-motor and supplementary motor area; IPFG, inferior prefrontal gyrus; STG, superior temporal gyrus; HA, hearing aid, ADHD, attention deficit/hyperactivity disorder; TD, typically developing.


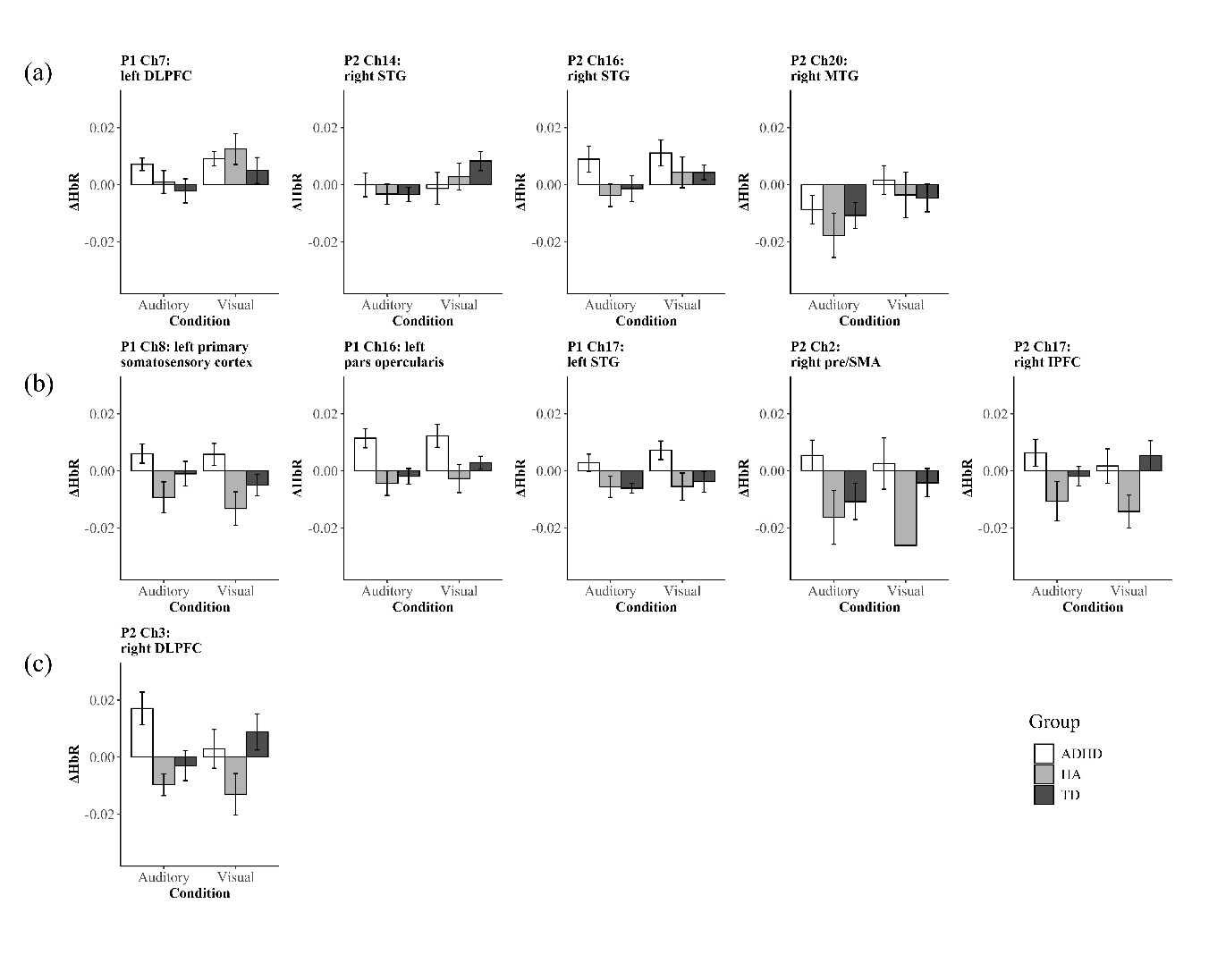


**Figure S3.** Effects of group (HA, ADHD, TD) and condition (auditory, visual) on ΔHbR. All effects of A. Condition: Visual > Auditory, B. Group and C. Group-by-condition interactions on ΔHbR are illustrated. Abbreviations: P, Probe set; Ch, channel; DLPFC, dorsolateral prefrontal cortex; MTG; medial temporal gyrus; pre/SMA, pre-motor and supplementary motor area; IPFG, inferior prefrontal gyrus; STG, superior temporal gyrus; HA, hearing aid, ADHD, attention deficit/hyperactivity disorder; TD, typically developing.

**Table S4.** Brain-behavior correlations

| **Group** | **Chromophore** | **Condition** | **Channel** | **Brain region** | **Statistics** |
| --- | --- | --- | --- | --- | --- |
| HA | - | - | - | - | - |
|  |  |  |  |  |  |
| ADHD | HbR | visual | P2 Ch20 | right MTG | *p*=0.04, *r_s_*= 0.53 |
|  | HbR | visual | P1 Ch8 | left primary somatosensory cortex | *p*=0.02, *r_s_*=0.63 |
| TD | HbO | auditory | P1 Ch6 | left DLPFC | *p*=0.01, *r_s_*=-0.66 |
|  | HbR | visual | P2 Ch3 | right DLPFC | *p*=0.007, *r_s_*=0.70 |

Note: Spearman correlations per group (HA, ADHD, TD) and condition (auditory, visual) are listed. Abbreviations: HA, hearing aid, ADHD, attention deficit/hyperactivity disorder; TD, typically developing; HbR, deoxygenated hemoglobin; HbO, oxygenated hemoglobin; P, Probe set; Ch, channel; MTG; medial temporal gyrus; DLPFC, dorsolateral prefrontal cortex.
