# Supplementary material for: Auditory and Visual Response Inhibition in Children with Bilateral Hearing Aids and Children with ADHD
Source: Brain Sci. 2020 May 18;10(5):307. doi: 10.3390/brainsci10050307 (PMC7287647; doi:10.3390/brainsci10050307)
Supplement: Supplementary file 1 [file brainsci-10-00307-s001.zip › SupplementaryMaterial_BrainSciences_LB.pdf]

Supplementary Material

Table S1. Hearing aid (HA) information for the HA group.

**HA user**	**Age at HL diagnosis: (years of age)**	**Age at first HA fitting: *M* (*SD*) (years of age)**	**HA use**		**Unaided pure-tone average threshold (0.5-4 kHz)**	
			**School day (h)**	**Out of school day (h)**	**Left ear (dB)**	**Right ear (dB)**
A			13	13	55	51.9
B			11	10	63.5	61
C			14	14	65.5	73
D			12	12	49	44.5
E			15	12	50	50.5
F			10	8	36.5	35.5
G			12.5	11	44.5	43
H	For 9 children < 2.5, for 4 children between 3-4, and for 2 children between 5-9 years of age	2.85 (2.29)	13	15	75	75
I			12	14	62	57
J			14.5	14.5	98	97
K			14.5	14.5	70	72
L			14	16	96	80
M			12	12	66	75
N			15.5	15.5	105	91
O			12.5	12.5	71	76

Note: The demographic data for the HA group is listed. For information on age, descriptive information and a group average are presented to ensure anonymity. The audiometry indicated an average hearing loss in children with HAs as follows: right ear: 55.33 dB for 0.25 kHz, 59 dB for 0.5 kHz, 63.5 for 1 kHz, 73.67 for 2 kHz, 69.46 for 3 kHz, 63 dB for 4 kHz, 66.43 for 6 kHz and 66.43 for 8 kHz; left ear: 51.33 dB for 0.25 kHz, 59.67 dB for 0.5 kHz, 65.67 for 1 kHz, 75.67 for 2 kHz, 68.08 for 3 kHz, 62.14 dB for 4 kHz, 67.14 for 6 kHz and 71.07 for 8 kHz. For an illustration of the audiometry see Figure S1. Abbreviations: HA, hearing aid/child (A-O) of the hearing aid group; M, mean; SD, standard deviation.

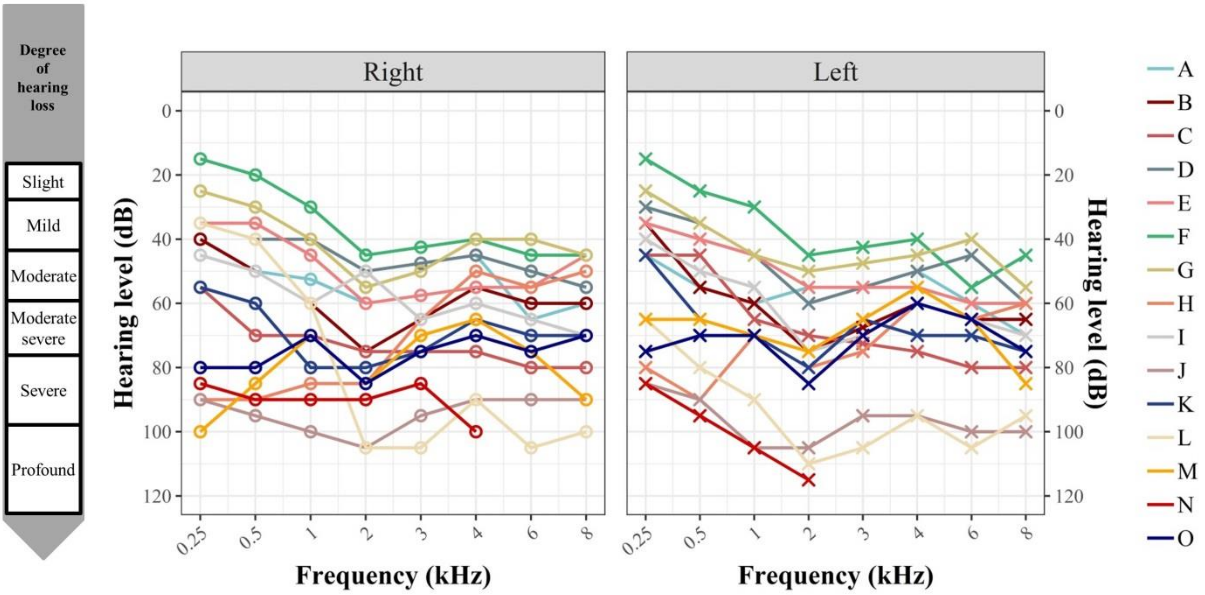

Figure S1. Unaided pure tone audiometry (air-conduction) of the right and left ear for each child (A-O) of the HA group.

Table S2. Uncorrected t-tests of the significant ($p < 0.05$) contrasts of ΔHbO in each condition against baseline.

	Ch	Group	Brain region	*t*	df	*p*
Visual	P1 Ch 4	ADHD	Left supramarginal gyrus	3.53	13	0.004
	P1 Ch 7	TD	Left DLPFC	-2.82	14	0.01
	P1 Ch 8	TD	Left primary somatosensory cortex	2.37	14	0.03
	P2 Ch1	ADHD	Right supramarginal gyrus	2.55	11	0.03
	P2 Ch2	HA	Right pre/SMA	2.43	10	0.04
	P2 Ch6	HA	Right primary somatosensory cortex	2.23	11	0.048
		ADHD		2.75	13	0.02
	P2 Ch9	ADHD	Right frontopolar cortex	2.35	14	0.03
	P2 Ch10	ADHD	Right primary auditory cortex	2.80	13	0.02
	P2 Ch15	TD	Right STG	2.57	14	0.02
	P2 Ch13	ADHD	Right frontopolar cortex	2.39	12	0.03
	P2 Ch16	TD	Right STG	-2.39	13	0.03
	P2 Ch17	HA	Right IPFG	3.43	9	0.008
	P2 Ch21	TD	Right temporopolar cortex	-2.73	14	0.02
Auditory	P1 Ch8	ADHD	Left primary somatosensory cortex	2.23	12	0.046
	P2 Ch1	ADHD	Right supramarginal gyrus	3.17	11	0.009
	P2 Ch5	ADHD	Right supramarginal gyrus	2.60	12	0.02
	P2 Ch6	ADHD	Right primary somatosensory cortex	4.17	13	0.001
	P2 Ch10	ADHD	Right primary auditory cortex	2.79	13	0.02
	P2 Ch18	ADHD	Right frontopolar cortex	2.30	14	0.04
	P2 Ch21	TD	Right temporopolar cortex	-2.74	14	0.02

Abbreviations: HA, hearing aid; ADHD; attention deficit/hyperactivity disorder; TD, typically developing; P, probe; Ch, channel; IPFG, inferior prefrontal gyrus; STG, superior temporal gyrus; MTG, medial temporal gyrus; pre/SMA, pre-motor and supplementary motor area.

Table S3. Uncorrected *t*-tests of the significant ($p < 0.05$) contrasts of ΔHbR in each condition against baseline.

	**Ch**	**Group**	**Brain region**	***t***	**df**	***p***
**Visual**	P1 Ch7	HA	Left DLPFC	2.31	11	0.04
		ADHD		3.59	12	0.004
	P1 Ch8	HA	Left primary somatosensory cortex	-2.25	11	0.046
	P1 Ch12	ADHD	Left pre/SMA	3.42	14	0.004
	P1 Ch16	ADHD	Left pars opercularis	3.05	12	0.01
	P1 Ch17	ADHD	Left STG	2.21	14	0.04
	P1 Ch18	TD	Left STG	2.62	13	0.02
	P2 Ch2	HA	Right pre/SMA	-2.91	10	0.02
	P2 Ch9	TD	Right frontopolar cortex	2.24	14	0.04
	P2 Ch14	TD	Right STG	2.46	14	0.03
	P2 Ch16	ADHD	Right STG	3.42	12	0.03
	P2 Ch17	HA	Right IPFG	-2.47	9	0.04
**Auditory**	P1 Ch3	ADHD	Left pre/SMA	2.34	11	0.04
	P1 Ch7	ADHD	Left DLPFC	3.25	12	0.007
	P1 Ch16	ADHD	Left pars opercularis	3.42	12	0.005
	P1 Ch17	TD	Left STG	-3.57	14	0.003
	P2 Ch3	HA	Right DLPFC	-2.51	7	0.04
		ADHD		2.99	12	0.01
	P2 Ch20	HA	Right MTG	-2.30	10	0.04
		TD		-2.39	14	0.03

Abbreviations: HA, hearing aid; ADHD; attention deficit/hyperactivity disorder; TD, typically developing; P, probe; Ch, channel; IPFG, inferior prefrontal gyrus; STG, superior temporal gyrus; MTG, medial temporal gyrus; pre/SMA, pre-motor and supplementary motor area.

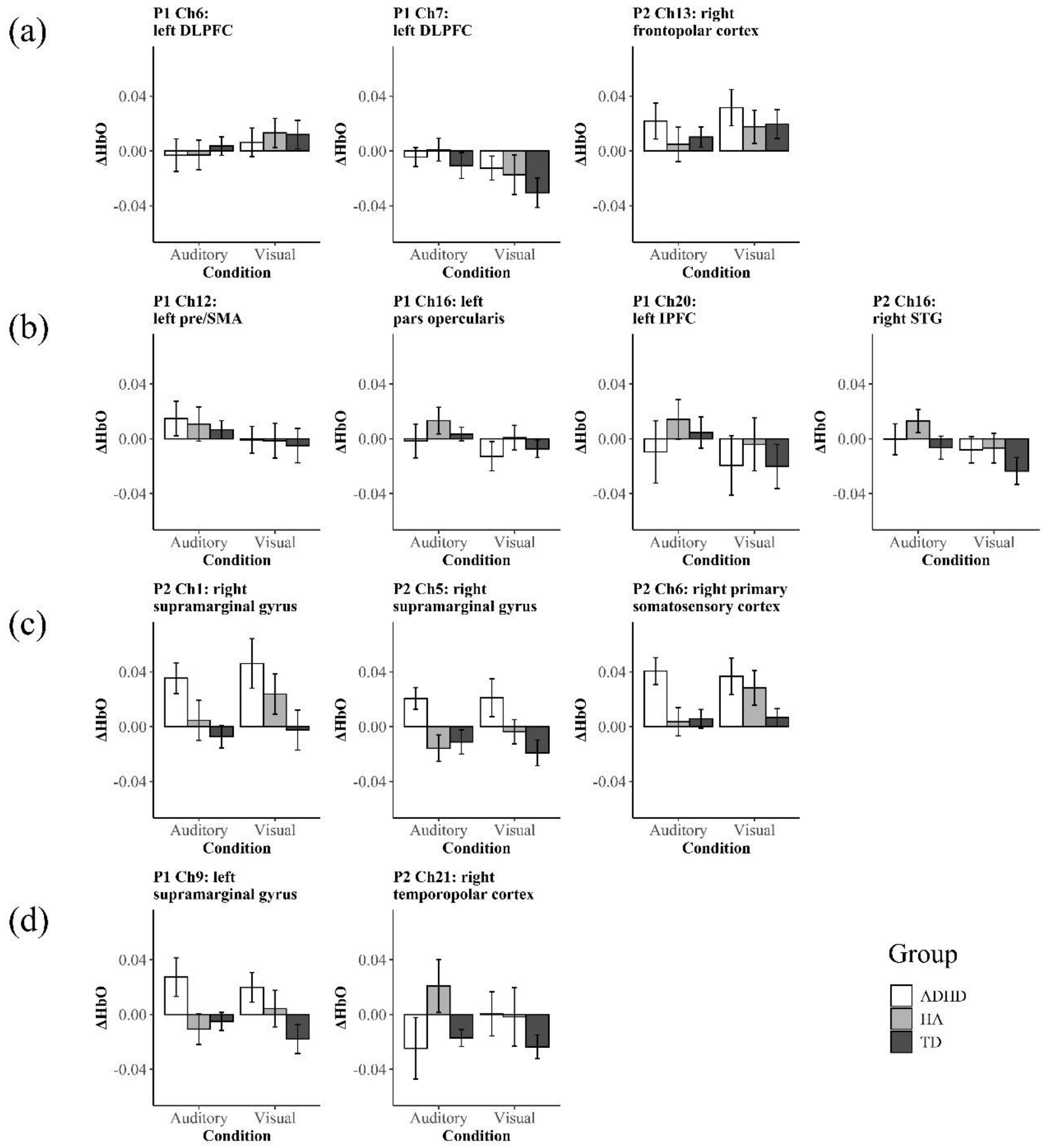

Figure S2. Effects of group (HA, ADHD, TD) and condition (auditory, visual) on ΔHbO. All effects of (a) Condition: Visual > Auditory, (b) Condition: Auditory > Visual, (c) Group and (d) Group-by-condition on ΔHbO are illustrated. Abbreviations: P, Probe set; Ch, channel; DLPFC, dorsolateral prefrontal cortex; pre/SMA, pre-motor and supplementary motor area; IPFG, inferior prefrontal gyrus; STG, superior temporal gyrus; HA, hearing aid, ADHD, attention deficit/hyperactivity disorder; TD, typically developing.

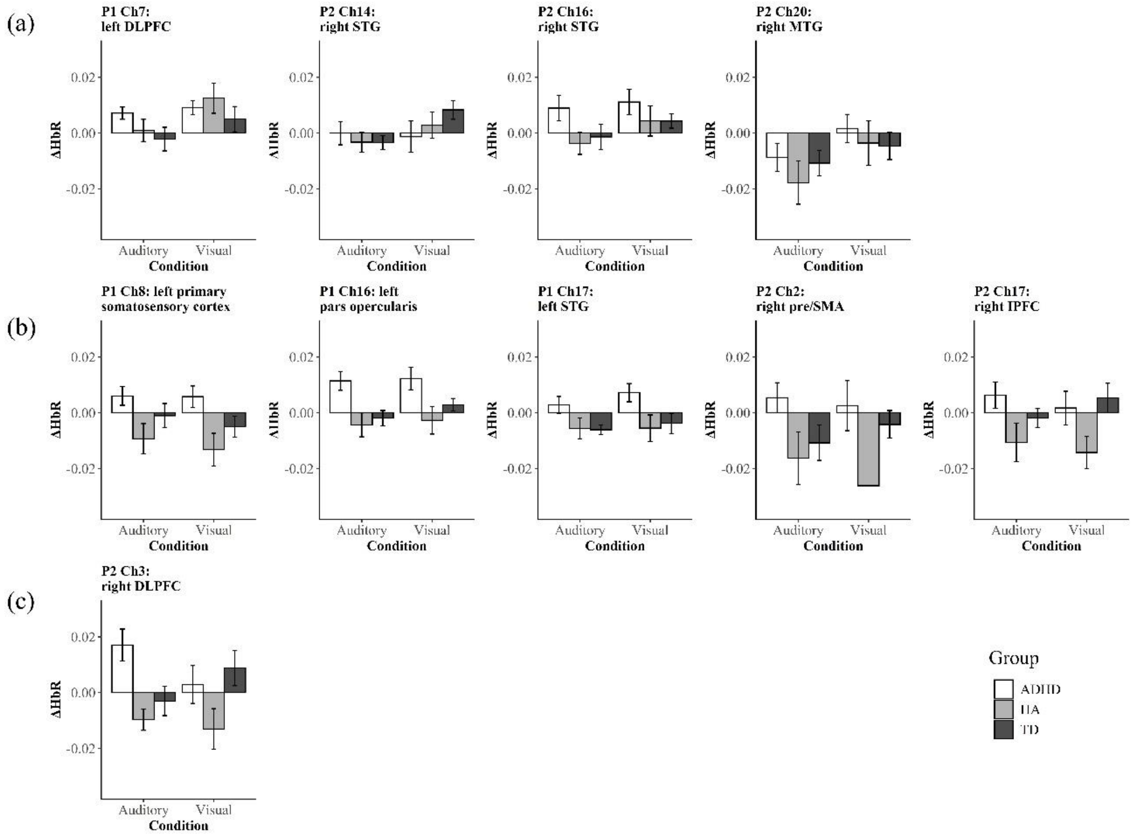

Figure S3. Effects of group (HA, ADHD, TD) and condition (auditory, visual) on ΔHbR. All effects of A. Condition: Visual > Auditory, B. Group and C. Group-by-condition interactions on ΔHbR are illustrated. Abbreviations: P, Probe set; Ch, channel; DLPFC, dorsolateral prefrontal cortex; MTG; medial temporal gyrus; pre/SMA, pre-motor and supplementary motor area; IPFG, inferior prefrontal gyrus; STG, superior temporal gyrus; HA, hearing aid, ADHD, attention deficit/hyperactivity disorder; TD, typically developing.

Table S4. Brain-behavior correlations

Group	Chromophore	Condition	Channel	Brain region	Statistics
HA	-	-	-	-	-
ADHD	HbR	visual	P2 Ch20	right MTG	p=0.04, r_s= 0.53
	HbR	visual	P1 Ch8	left primary somatosensory cortex	p=0.02, r_s=0.63
TD	HbO	auditory	P1 Ch6	left DLPFC	p=0.01, r_s=-0.66
	HbR	visual	P2 Ch3	right DLPFC	p=0.007, r_s=0.70

Note: Spearman correlations per group (HA, ADHD, TD) and condition (auditory, visual) are listed. Abbreviations: HA, hearing aid, ADHD, attention deficit/hyperactivity disorder; TD, typically developing; HbR, deoxygenated hemoglobin; HbO, oxygenated hemoglobin; P, Probe set; Ch, channel; MTG; medial temporal gyrus; DLPFC, dorsolateral prefrontal cortex.
